# A dataset on human perception of and response to wildfire smoke

**DOI:** 10.1038/s41597-019-0251-y

**Published:** 2019-10-24

**Authors:** Mariah Fowler, Arash Modaresi Rad, Stephen Utych, Andrew Adams, Sanazsadat Alamian, Jennifer Pierce, Philip Dennison, John T. Abatzoglou, Amir AghaKouchak, Luke Montrose, Mojtaba Sadegh

**Affiliations:** 1 Department of Civil Engineering, Boise State University, Boise, Idaho 73725 US; 2 School of Public Service, Boise State University, Boise, Idaho 83725 US; 3 College of Business and Economics, Boise State University, Boise, Idaho 83725 US; 4 Department of Geosciences, Boise State University, Boise, Idaho 83725 US; 5 Department of Geography, University of Utah, Salt Lake City, Utah 84112 US; 6 Department of Geography, University of Idaho, Moscow, Idaho 83844 US; 7 Department of Civil and Environmental Engineering, University of California, Irvine, California 92697 US; 8 Department of Community and Environmental Health, Boise State University, Boise, Idaho 83725 US

**Keywords:** Decision making, Government, Education

## Abstract

Wildfire smoke presents a growing threat in the Western U.S.; and human health, transportation, and economic systems in growing western communities suffer due to increasingly severe and widespread fires. While modelling wildfire activity and associated wildfire smoke distributions have substantially improved, understanding how people perceive and respond to emerging smoke hazards has received little attention. Understanding and incorporating human perceptions of threats from wildfire smoke is critical, as decision-makers need such information to mitigate smoke-related hazards. We surveyed 614 randomly selected people (in-person) across the Boise Metropolitan Area in Idaho and 1,623 Boise State University affiliates (online), collecting information about their level of outside activity during smoke event(s), knowledge about the source of air quality information and effective messaging preference, perception of wildfire smoke as a hazard, and smoke-related health experiences. This relatively large dataset provides a novel perspective of people’s perception of smoke hazards, and provides crucial policy-relevant information to decision-makers. Dataset is available to the public and can be used to address a wide range of research questions.

## Abstract (summary)

**Table:** 

Measurement(s)	Perception • response to
Technology Type(s)	survey method
Factor Type(s)	hazard condition • age • sex • income • education
Sample Characteristic - Organism	Homo sapiens
Sample Characteristic - Environment	wildfire • smoke
Sample Characteristic - Location	Ada County

Machine-accessible metadata file describing the reported data: 10.6084/m9.figshare.9938252

## Background & Summary

Area burned by fires and the length of fire season have increased across much of the Western U.S. in recent decades^1–5^. Anthropogenic climate change has significantly increased fire-season fuel aridity that promotes favourable fire conditions^6^. Humans also expanded the fire niche by igniting 60% of fires over the Western U.S. between 1992–2012^7^, and impacted fire regimes through fire suppression^8^. This increased wildfire activity has degraded fire-season air quality in the Western U.S.^9–11^; which in turn increased mortality and induced aggravated respiratory, cardiovascular, mental and perinatal health issues^12–14^, as well as secondary economic impacts such as recreation and tourism^15^.

The growing wildfire smoke hazard in the Western U.S. demands attention from policy- and decision-makers to reduce associated risks. Given projections for future warming (and future increases in wildfire activity in the Western U.S.^16^; also see Andela^17^), it is likely that fire and smoke will be part of the western landscape for decades to come. Hence, developing adaptation and mitigation plans for fire/smoke-prone areas is urgent, which requires an understanding of how people perceive this hazard and respond to it. Lack of region-specific social behavioural understanding may render “well-intended policies” ineffective^18^. While a majority of traditional risk-related literature is focused on the natural drivers of the hazard^19,20^, the human role in responding, or not, to hazards is increasingly gaining attention^21^. Humans’ response to hazard is dependent on their interpretation of the risk, which is “*shaped by their own experience*, *personal feelings and values*, *cultural beliefs and interpersonal and societal dynamics*”^22^.

The literature on the social behavioural aspect of wildfire smoke risk mitigation is sparse^23–25^. One line of research in this field is focused on assessing effective public health messaging^26–28^. Messages should be short, direct and clear^29–32^, context-specific and inclusive^33^, should address at-risk population concerns^34^, and should be issued by trusted institutions^35^. Moreover, different strata of the public have distinct favourable communication channels^36–38^. Another line of research in this field is focused on the public acceptance of smoke generated from different sources and perception of it as a hazard^39–41^. Although significant strides have been made in this field, social behavioural understanding of response to wildfire smoke hazard is not fully realized.

This paper introduces a dataset that can help bridge a knowledge gap in the literature on how people in the Western U.S. respond to wildfire smoke. This area and much of the interior Northwestern U.S. has experienced significant increases in poor air quality episodes over the past three decades due to wildfire smoke^9^, which is manifested through widespread impacts on human health and economic burden^14^. This dataset is intended to contribute to understanding human response to and perception of wildfire smoke. We investigate (i) the channels through which people receive air quality information, (ii) effective public health messaging content and timing that affect people’s response to smoke events, (iii) public perception of smoke as a hazard, and (iv) associated health issues, and measures taken to mitigate the negative health experiences. The questionnaire also gathers demographic, current health, and activity information to help contextualize human response to the hazard based on background. Data is gathered through online and in-person surveys. The in-person mode of data collection targets the population that engage in outdoor activities – as they receive the highest dose of smoke – and the elderly – who might not have access or would not participate in an online survey. The online survey covers a wider range of age demographics. Information from a total of 614 in-person and 1,623 online participants have been assembled in this dataset^42^.

## Methods

### Questionnaire design

Our survey questionnaire includes five categories of questions: demographic data, activity data, air quality notification, natural hazard, and health. These categories were carefully selected to cover different aspects of social behavioural studies on the wildfire smoke hazard^25,41,43^. Furthermore, we held several in-person and virtual meetings with the federal, tribal and regional partners to identify the data that could support stakeholders decision making and could inform wildfire smoke mitigation strategies. Questions and their associated options were then designed according to the literature (details later) and the needs of the stakeholders. The questionnaire was then refined by our team members in close collaborations with the Idaho Department of Environmental Quality, Bureau of Land Management (Boise office) and Nez Perce Tribe to ensure the wording was neutral and the questions/options resonated with the needs of the decision-makers. Table 1 summarizes the questions, and the complete questionnaire is provided in Appendix A. Moreover, a detailed version of this table with response rates for each question is provided in the Supplementary Information, SI (Table S1). Our comprehensive questionnaire provides critical information to elucidate how one person’s background and experiences translate to a certain belief or behaviour.

**Table 1:** Summary of survey questions for 614 in-person and 1,623 online participants.

Category	Questions content
Demographic Data (6 questions)	• Age, • Gender, • Race, • Zip code, • Education level, • Income
Activity Data (3 questions)	• General health status, • Engagement in outside activities, • Frequency of outside activities
Air Quality Notification (13 questions)	• Receiving/Seeking air quality information and its source, • Frequency of seeking air quality information, • Reducing outside activities, • Longest period of consecutive days to reduce outside activities, • Minimum air quality index that convinced to reduce/eliminate outside activities, • Effective warning content and delivery method, • Timing of warning, • Future mitigation planning
Natural Hazard Questions (3 questions)	• Perception of smoke as a hazard, • Comparison with other hazards such as tornadoes and hurricanes, • Evacuating home to prevent smoke impacts
Health Questions (3 questions)	• Smoke-related health experience, • Type of observed symptoms, • Mitigation strategies to reduce health issues

**Fig. 1:**
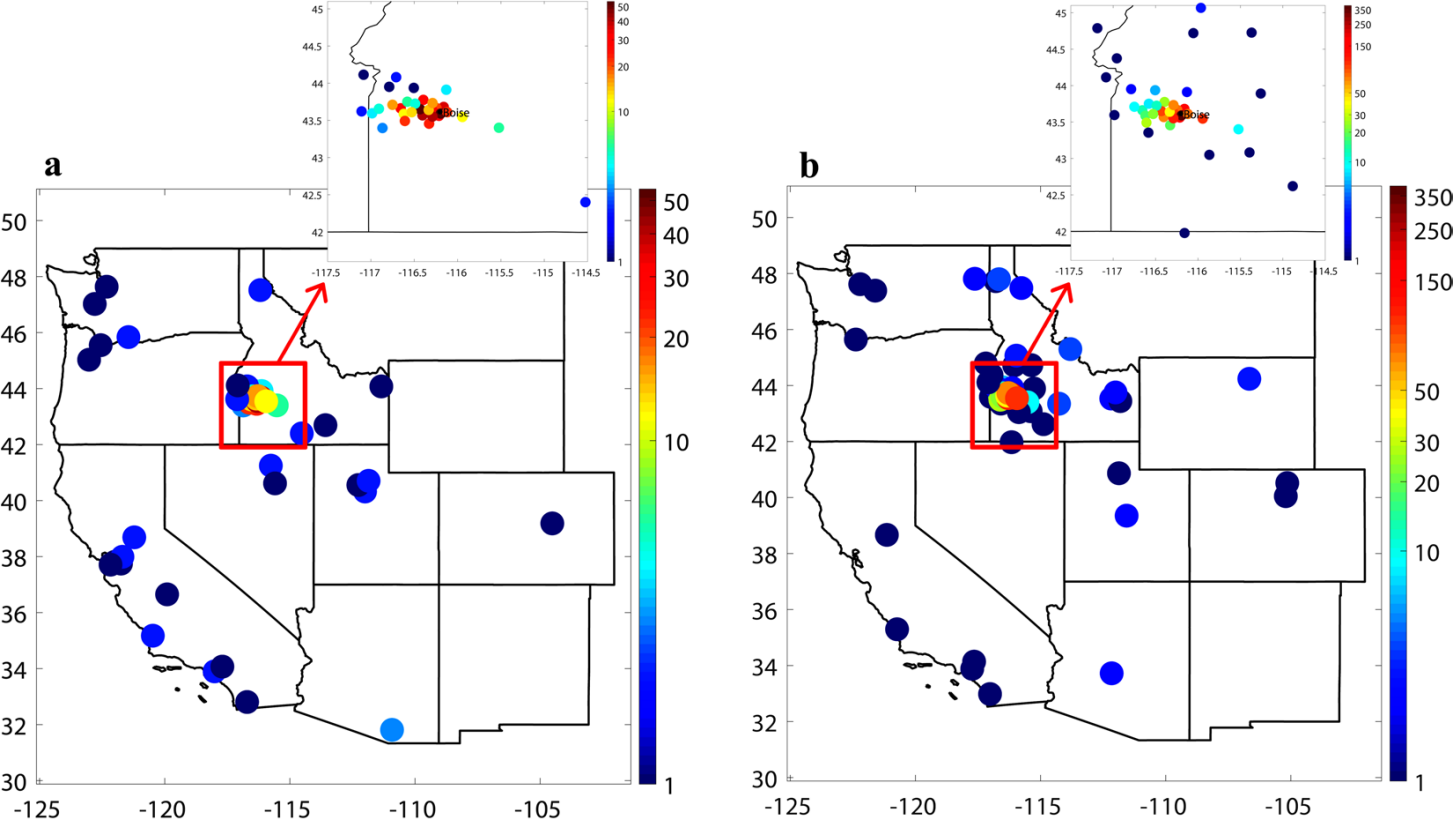
Spatial distribution of residence zip codes identified by survey participants. (**a**) In-person participants. (**b**) Online participants. Frequency of the collected samples in each location is color-coded in log-scale.

This questionnaire was created in accordance with best practices across social science disciplines. To the extent possible, we used wording that mirrored questions on national social science studies^44–48^, such as the American National Election Study and General Social Survey. Demographics were collected to be most useful to a broad scope of researchers, measuring key social and economic characteristics of respondents. Questions were further designed to minimize ambiguity – the questionnaire used branching questions, when applicable, to allow participants to select a simple “yes” or “no” answer, before being asked a follow-up question to provide more detail on their answer. When multiple options were likely to apply to an individual, participants were asked to select all choices that apply to them, and provided an “other” option if they chose to volunteer another answer. For questions that were potentially ambiguous or difficult to answer, respondents were provided with an explicit “Not sure” response option (see Tourangeau^49^). Respondents were allowed to skip any question they did not feel comfortable answering. Questions directly related to smoke events mentioned the summer of 2018 time period, so participants were all able to consider the same time period when responding to the survey. Given the intensity of the smoke event in summer 2018 in the Boise Metropolitan Area in Idaho (Figs S1–S5), we expect that most respondents recalled this event. Note that smoke events are regional, covering hundreds of thousands of square kilometres, and the smoke-impacted area in the Western U.S. in 2018 coincides with the self-reported residence zip codes of our participants (compare Fig. 1 with Fig. S6).

Anecdotally, the survey team observed that this questionnaire was relatively straightforward for respondents to answer (see Technical Validation section for steps taken to ensure questions’ clarity). Respondents selected an answer for the vast majority of questions (Table S1), and required little clarification of the questions in the in-person surveys. Most respondents did not seek clarification about the smoke event, suggesting that it was a salient event in respondents’ memories.

### Modes of surveying

We surveyed 614 people in-person in different areas of the Boise Metropolitan Area in Idaho (including cities of Boise, Eagle, Caldwell, Kuna, Meridian and Nampa) between August 28 to September 15, 2018. An online survey was also emailed to 8,768 Boise State University students, faculty and employees, of which 1,623 completed the survey between September 25 to October 16, 2018. This dataset includes information from a large group of at-risk population (elderly and those with pre-existing conditions), including 55 individuals older than 65 years and 45 participants with “Fair” or “Poor” health status in our in-person survey. In the online survey, at-risk population is even larger with 65 participants over 65 years old and 157 individuals with “Fair-Poor” health condition. We also have a relatively large group of Hispanic/Latino participants (35 in-person and 99 online participants) and moderate- to low-income population, defined as **household** income of less than $50,000 per year (185 in-person and 578 online participants).

We adopted two methods of surveying: in-person and online. While large population of participants in each survey mode permits separate analysis, their cross-comparison can also provide valuable information about the behavioural preferences of the two groups (more in Usage Notes). These two modes target a wide and complementary spectrum of demographics. A great portion of the online participants are 18–22 years old, while the in-person surveys focus on the older generations that might not have access to the internet or might not be comfortable with taking an online survey. Given the nature of this study, we have a great interest in the preferences of the outgoing elderly, who are most at risk due to wildfire smoke. The in-person section of this dataset^42^ is unique as a portion of the participants in this study (mainly, but not limited to, elderly) would not engage in any online survey due to lack of access or familiarity with the internet and/or potential mistrust to the data gathering entities – which is not the case for Boise State affiliates due to the deep connection of the university to the community.

### In-person survey

We conducted the in-person surveys between August 28 and September 15, 2018 in several public locations, as allowed by the Institutional Review Board (IRB) permit, across the Boise Metropolitan Area in Idaho, including Boise, Meridian, Nampa, Eagle, Kuna and Caldwell. We have taken all efforts to diversify the population to the extent possible. Moreover, one of our goals was to approach people who regularly engage in outdoor activities, as they receive highest dose of smoke, to understand their social behavioural response to wildfire smoke. This was achieved as roughly 95% of our survey population engaged in outdoor activities. Table S2 provides details of the locations and times that answers were collected.

Collected paper surveys were then converted into digital format by 8 volunteer undergraduate students, 5 volunteer graduate students, and 6 other volunteers. Each data entry person was assigned 20–50 paper surveys. Each survey paper was tagged with a number in X-Y format, in which X is the batch number (survey collected at each location are one batch) and Y is the survey paper number in this batch. To ensure the quality of this transition, each batch has been double-checked twice (more information available in Technical Validation). A scanned version of all paper surveys are also provided along with the digital version of the dataset^42^ for interested audience.

### Online survey

**Fig. 2:**
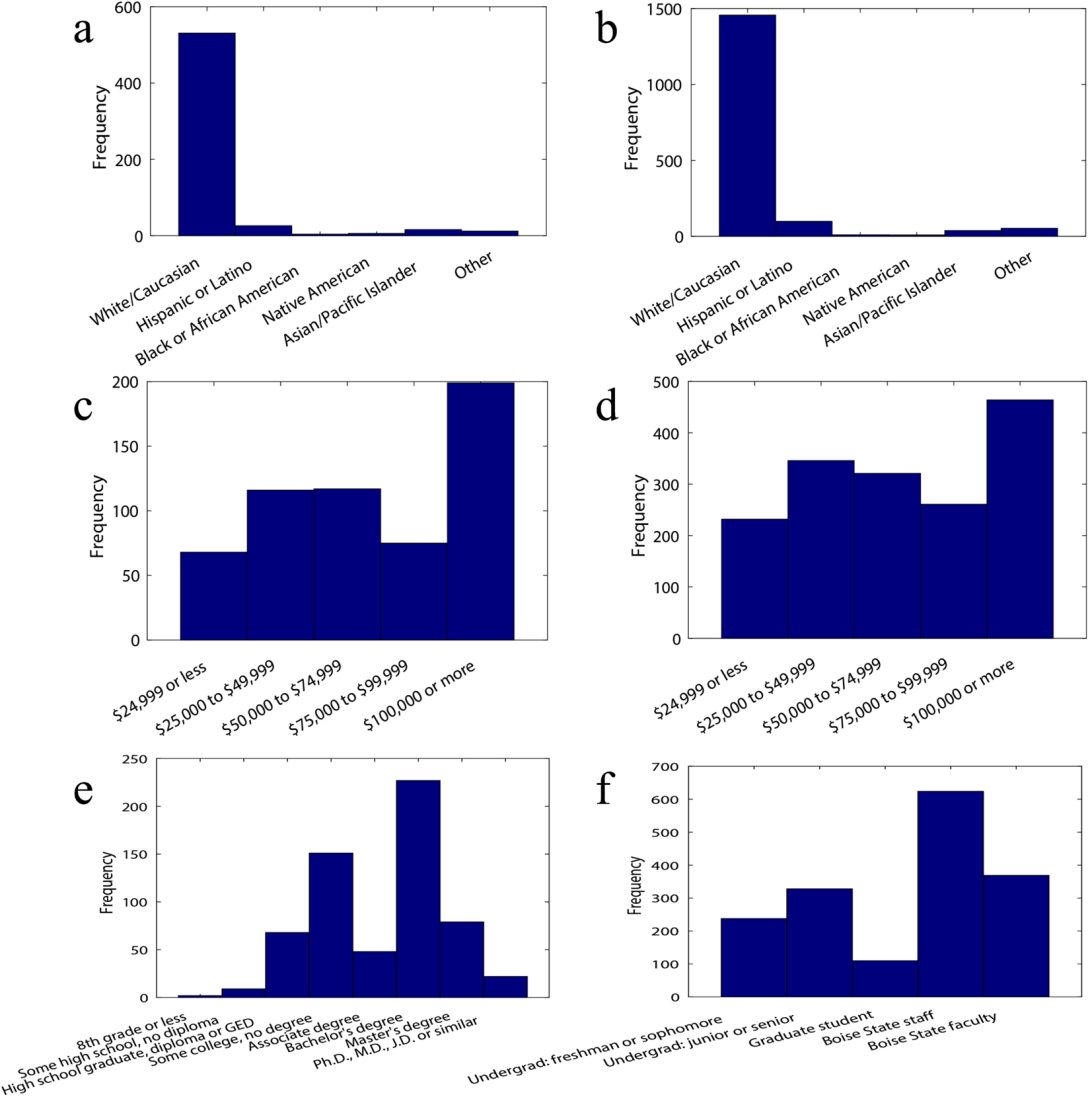
Socioeconomic background of participants. (**a**,**b**) Racial, (**c**,**d**) household income level and (**e**,**f**) education level distribution of participants in in-person (**a**,**c**,**e**) and online (**b**,**d**,**f**) surveys.

An online version of the questionnaire was sent to a randomly selected subset (5,020) of Boise State students as well as all faculty and staff (3,748) through Qualtrics experience management service. The first email was sent on September 25, 2018, followed by two reminder emails to those who did not respond in the first round on October 1 and October 3, 2018. A total of 1,623 completed responses were collected between September 25, 2018 and October 16, 2018. Figure 2f provides more details on the faculty, staff and student distribution of online participants. The online collected surveys expand the age demographics of participants and include a large 18–22 years old population.

### Ethics board review and informed consent

This study was approved by the Social & Behavioural Review Board (SB-IRB) of Office of Research Compliance (ORC) of Boise State University on September 18, 2018. Protocol number for this study is 126-SB18-156. All participants were at least 18 years old, and consented to take part in this study. Background information about the goals of the study, time required to fulfil the questionnaire, potential risks, and contact information of the principal investigator (Mojtaba Sadegh) and Internal Review Board (Office of Compliance) of Boise State University were provided to the participants before asking about their consent. Participants were given the choice to keep the cover letter that provided the study background and principal investigator’s contact information. The cover letter highlights potential risks to participants as:

*“This study involves no foreseeable risks*. *You may discontinue the study at any time*. *Your responses are completely anonymous and cannot be linked to you in any way*.

*For this research project*, *we are requesting demographic information*. *Though it is unlikely*, *it is possible that the combined answers to these questions may make an individual person identifiable*. *The researchers will make every effort to protect your confidentiality*. *However*, *if you are uncomfortable answering any of these questions*, *you may leave them blank”*.

Questions do not ask for any identification information and are designed to keep the anonymity of the participants. Please see Appendix A for more details provided to participants.

## Data Records

This dataset is provided in a CSV format along with its metadata in Extensible Markup Language (XML) and text formats. Each row in the data file represents one participant and each group of columns represent one question (clearly marked). Each column represents one option and is assigned a binary value, in which “1” illustrates selecting and “0” signifies not selecting that option. For example, question 8 asks about general health status of the participant and has 4 options: 1. Excellent, 2. Good, 3. Fair, 4. Poor. This question is assigned 4 columns with each column representing one of the health conditions. If “Fair” was selected by the participant, column 3 in this group is assigned 1 and others are assigned 0. Other questions might allow for multiple selections, in which case more than one column can be assigned 1. A metadata file accompanies the dataset and provides details of the questions and the options. Note that in-person and online survey results are provided in two separate files to mark their difference clearly, as they were conducted in different modes, among different populations, and at different times. The metadata with full description of its elements is provided within an Extensible Markup Language (XML) file, which is intended to extend a standard way for programmers and others to use the provided information. Metadata is also provided in a “README” file in text format. This dataset^42^ is freely available to public.

Here, we present demographic information collected from in-person and online surveys. This data informs the future subsampling efforts (see Technical Validation section).

**Fig. 3:**
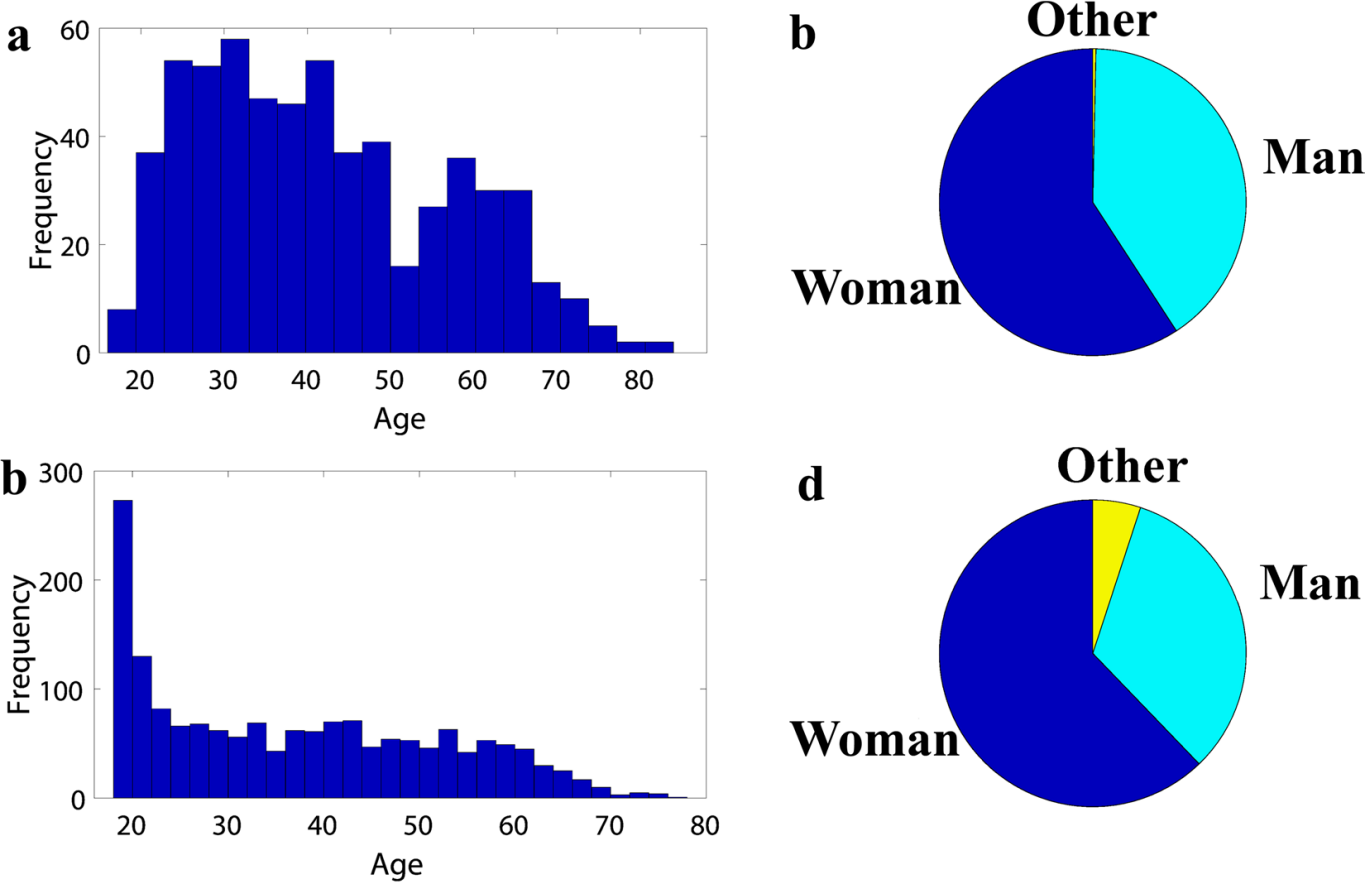
Demographic of survey participants. (**a**) Age and (**b**) gender distribution of in-person survey participants. (**c**) Age and (**d**) gender distribution of online survey participants.

Figure 1 displays the spatial distribution of the residence location of participants. Expectedly, a majority of the participants both in in-person (Fig. 1a) and online (Fig. 1b) surveys are residents of Idaho. Online participants (Fig. 1b; Boise State community) identified their residence location from a more widespread portion of Idaho, as compared to in-person surveys, which is also anticipated given the student populous in the online survey. This figure shows that the majority of the participants of this study reside in smoke prone area of the interior Northwest. Due to the regional nature of the smoke events, the timing of the surveys, and the concentration of respondents in Idaho, we believe that the vast majority of respondents would have observed a smoke event (see Fig. S6). Indeed, as Fig. 3 of McClure^9^ shows extreme tail of particulate matter distribution (poor air quality intensity) shows an increasing trend in much of the Northwest, which coincide with the residence zip code of an absolute majority of this study participants (Fig. 1). Note that although in-person survey was conducted in the Boise Metropolitan Area, Idaho, some participants from across the western U.S. visiting Boise also engaged in our survey (Fig. 1a).

Figure 2 displays racial, income and education level distributions of the participants in this study. As anticipated, a majority of the participants in this study are white, which is representative of the population of Boise Metropolitan Area in Idaho (see Table S3; also see Ada County, which constitutes majority of Boise Metropolitan Area’s population, census facts at https://www.census.gov/quickfacts/adacountyidaho). A great portion of the participants (35% of in-person and 29% of online survey participants) reported $100,000 or more **household** income. Moreover, highest portion of in-person survey participants hold a bachelor’s degree, and Boise State staff constitute the largest portion of online participants.

Figure 3 shows that a majority of in-person survey participants were in their 20 s to 40 s, whereas a great portion of people who took the online survey were between 18 to 22 years old, as expected given the college student population at Boise State University. This figure also shows that the majority of the people that took the survey were women.

## Technical Validation

There are two steps required before and after conducting a survey to minimize various possible sources of uncertainty and/or error. These steps are adopted from the literature^50–54^ to ensure implementation of the right methodology, warrant quality of the collected data, secure correctness of post-processing, and ultimately assure the appropriateness of analysis:

1. Before conducting the survey: A new questionnaire was designed based on best practices across social science discipline. The team that contributed to the design of the survey questionnaire includes experts from regional (Idaho Department of Environmental Quality), tribal (Nez Perce Tribe) and federal (Bureau of Land Management and Environmental Protection Agency) governments as well as academia to ensure questions are responsive to the needs of a broad audience both from the scientific community and the practitioners. To the extent possible, this survey used wording that mirrored questions on national social science studies.
  Face validity^55,56^ was established by two groups of individuals. The first group consisted of ten individuals without technical background that assessed the clarity of the question to the public. This group were also tasked to identify technical words that might not be intelligible for the public. The second group consisted of five social scientist experts who technically judged the construction of questionnaire, further ensuring that the questions were not vague or misleading.
2. After conducting the survey: Paper survey data entry into digital format, unless automated, is always associated with human errors (e.g. entering option 3 instead of 2 when digitizing paper surveys). We divided the paper surveys into batches of 30–50, and assigned them to one person to enter to excel format. Each person and each batch were assigned identifier codes. We re-evaluated data entry for each batch twice by two separate people. Each time, we randomly evaluated 10% of each survey batch for data input errors. The detection of any errors in each sample required the entire population of the batch to be double-checked and corrected, if necessary.

The questions with open-ended options, such as “other”, are specifically challenging given answers can vary widely and spelling/typing errors can complicate the categorization process. We devised an algorithm to find all new words (including typing errors) and one person manually clustered them into groups with similar characteristics, which are then assigned numeric codes. The metadata includes all participant-used words that are clustered under each category. Note that all options are presented in binary format, with “1” representing selecting and “0” representing not selecting that option.

A major source of uncertainty in this dataset^42^ is the adopted convenience sampling method. We have surveyed people in public areas, mostly parks and public events, and also surveyed the Boise State community (online), which might pose some bias for the future analyses of this data as a result of under- or over-sampling a strata of the public. We strongly recommend that future users subsample the dataset using the demographic information to fit their study needs. Tables S3–S5 in the Supplementary Information provide age, education and race demographics of Boise, Idaho, according to the 2017 American Community Survey and provide sources to inform future subsampling efforts, if the purpose is to analyse the perceptions and responses of the entire community to wildfire smoke. These tables also provide general statistics about the demographics of our survey participants. We also encourage using an ensemble of subsamples to assess uncertainty ranges of any analysis given the study goals and scientific hypotheses.

The survey team took care in the design and implementation of this study, and expect that errors in survey response are random, and not correlated with demographic or personal characteristics. Most potential errors are largely impossible to detect, but theory and previous research does not give us reason to expect that individual characteristics like age, gender, race and education are likely to impact knowledge of the wildfire smoke event, given its intensity and recency in the limited geographic area of the study. Of course, this does not mean the study is without potential for error. Individuals may struggle to place themselves on a response “scale”, and the response scale may vary for various individuals–for example, one person’s threshold for “strongly agreeing” with a statement may be different from another person’s^57^. The “scaling” issue, however, is expected to average out given the large population size of this dataset^42^. Another potential source of error, at least in the paper survey, is the formatting of the survey. To reduce waste, the surveys were printed on both sides of sheets of paper. This might have affected response rates for questions on the backside of the questionnaire. We do not find any systematic problems with non-response bias due to the side of the paper the question was printed on (see Table S1), but appreciate this is a possible source of uncertainty/error. Additionally, the very last question was printed on the back of the last page, by itself. We are most concerned about non-response to this question. Note, however, that only 9% of the people who responded with observing a symptom associated with wildfire smoke (one to the last question) didn’t respond to adopting a strategy to help with the smoke symptoms (the last question). Not necessarily all that observed symptoms (such as eye irritation) would take medication or visit a hospital, thus we do not believe there is any systematic error in the responses to the last question either. Another potential limitation is that the survey does not specifically ask each participant whether they have experienced a smoke event, but given the scale of such events, we are confident this would not bias our results. Finally, we acknowledge that self-reported behaviours might be associated with a range of biases, including social desirability and memory distortions. Such biases, however, are less important when a large population is studied, as the individual positive, negative biases cancel out, and average response is expected to represent the “true” behaviour of the target community.

## Usage Notes

This dataset can answer a wide variety of scientific questions and provide valuable actionable information about social-behavioural aspects of wildfire smoke. A non-exhaustive set of questions include:

1. What is the most effective medium to communicate air quality notification and wildfire smoke warning? What time is most effective to reach out to the public?
2. What is the most effective message content to influence human response to wildfire smoke?
3. What is the minimum EPA air quality rating that affect people’s decision to reduce/eliminate their outside activities? How familiar are the participants with such rating?
4. How can future efforts support mitigation actions by the public to reduce wildfire smoke hazard?
5. What mitigation strategies (e.g. evacuation) should be included and excluded in devising action plans?
6. Is wildfire smoke a major public health threat?
7. What are the measures that people take to alleviate wildfire smoke symptoms?
8. Does the public consider wildfire smoke a hazard? If so, what is the extent that the public is willing to act to mitigate wildfire smoke hazard?
9. Do demographics affect rate of people who received/sought air quality notification? Does seeking air quality notification warrant taking action to mitigate smoke hazards?
10. Is the rate of willingness to take action to mitigate wildfire smoke hazard higher for people that previously experienced illnesses? What about those who experienced (minor) health symptoms?
11. Are those with pre-existing health conditions more sensitive to risks of wildfire smoke?
12. What demographic indicators (e.g. age, income, race) are most influential in developing predictive understanding of human behaviour in response to wildfire smoke (responses to questions 1–11)?

Future studies can investigate the difference between responses to these and a plethora of other questions that are available in the dataset across different age, gender, race, income and education spectra. We strongly recommend that future users of this dataset subsample this dataset given the demographic information to serve their study goals. For example, the online survey includes a large number of 18–22 years old participants, which does not represent the age distribution of Boise Metropolitan Area in Idaho. However, one can readily subsample this dataset to represent the age distribution of the area of interest or the target study. Additionally, the over-sample of 18–22 year old university students would allow for substantial subgroup analyses among them. Furthermore, we strongly recommend that future users of this data employ bootstrapping approach to minimize the bias that might be associated with some subsamples. This requires that several subsamples – each representing the target demographics – are randomly selected and statistical tests are repeated on each. Ensemble of test results provides the uncertainty range, and mean ensemble better represents the actual response than each subsample individually. The large sample size allows for bootstrapping and helps quantify uncertainty ranges for statistical tests.

This dataset also provides a useful tool for future researchers, especially in the social sciences. This will provide some evidence on what types of messaging is most effective at encouraging individuals to engage in behaviours that reduce risks to their health. It further provides a clear template for future researchers interested in studying environmental hazards, and provides clear avenues for cost-effective data collection among a geographically limited target population of interest, allowing researchers to collect a large dataset with limited resources.

### Illustrative analysis

As an illustrative example, we evaluate potential association between being impacted by smoke in terms of illness observed in the household and perception of wildfire smoke as a hazard. We construct contingency table (Table 2) from our dataset^42^, which breaks down the perception of hazard conditioned on illness experience. We then use this table to test the null hypothesis that there are no non-random associations between the two categorical variables (perception of hazard and illness experience), against the alternative hypothesis that there is a non-random association between the two. To test this hypothesis, we use Fisher’s exact test, which is a statistical significance test specifically suited for contingency tables^58^. We also use Cohen’s kappa^59^ to evaluate the inter-rater reliability (i.e. degree of homogenous rating by various groups).

**Table 2:** Contingency table that shows associations between wildfire smoke induced illness and perception of smoke as a hazard, as well as Fisher’s and inter-rater reliability tests’ results.

	In-person		Online	
	Hazard	Not hazard	Hazard	Not hazard
Illness observed	98	10	340	33
Illness not observed	336	39	731	63
**Fisher’s test:**				
p-value	0.86		0.65	
Odds ratio	1.14		0.89	
99% Confidence interval	0.44–2.97		0.50–1.58	
Null hypothesis	Accept (there is no association)		Accept (there is no association)	
**Inter-rater reliability test:**				
Observed agreement	0.28		0.35	
Random agreement	0.27		0.35	
Cohen’s kappa	0.0055		−0.0061	
95% Confidence interval	−0.0503–0.0613		−0.0480–0.0358	
Level of agreement	Slight agreement		Poor agreement	
p-value	0.95		0.91	
Null hypothesis	Accept (observed agreement is accidental)		Accept(observed agreement is accidental)	

The Fisher’s test cannot reject the null hypothesis that there is no non-random association between these two categorical variables at 1% significance level. While this does not necessarily prove that the relationship between the two variables is random, high p-values of 0.86 (in-person survey) and 0.65 (online) show we are too far from rejecting the null hypothesis. Odds ratio for the in-person survey data is 1.14 (99% confidence interval: 0.44–2.97) and for the online surveys is 0.89 (99% confidence interval: 0.50–1.58), which also attest to the lack of a statistically significant association between the two categorical variables. While Fischer’s test generally points to no association between observing illness and perceiving smoke as a hazard (people generally perceive smoke as a hazard regardless of observing an associated illness), a more detailed inter-rater reliability analysis using Cohen’s kappa coefficient^59^ shows that the agreement between perceiving smoke as a hazard and observing/not-observing illness is accidental. Indeed, there are only slight and poor agreements between these two categorical variables among in-person and online survey participants, respectively. While hypothesis testing is beyond the scope of this study, this analysis serves as an example on how this data could be used. Note, however, to draw un-biased and robust inferences, one should select a subsample of this data that is representative of the entire population in Boise Metropolitan Area. Also, note that both these variables include an option “Not sure”, which is excluded from the analysis.

## Supplementary information

Supplementary Information.

Appendix A.

Appendix B.

## Data Availability

No computer program was used to generate this dataset^42^. Data was collected through in-person paper-based survey and online Qualtrics-based experience management service, both of which translated into consistent CSV format dataset.
