# Supplementary material for: A dataset on human perception of and response to wildfire smoke
Source: Sci Data. 2019 Oct 24;6:229. doi: 10.1038/s41597-019-0251-y (PMC6813346; doi:10.1038/s41597-019-0251-y)
Supplement: Supplementary file 1 — Supplementary Information. [file 41597_2019_251_MOESM1_ESM.docx]

**Supplementary Information for**

**A dataset on human perception of and response to wildfire smoke**

Mariah Fowler^1^, Arash Modaresi Rad^1^, Stephen Utych^2^, Andrew Adams^1^, Sanazsadat Alamian^3^, Jennifer Pierce^4^, Philip Dennison^5^, John T. Abatzoglou^6^, Amir AghaKouchak^7^, Luke Montrose^8^, Mojtaba Sadegh^1^

^1^Department of Civil Engineering, Boise State University, Boise, Idaho 73725

, U.S.

^2^School of Public Service, Boise State University, Boise, Idaho 83725, U.S.

^3^College of Business and Economics, Boise State University, Boise, Idaho 83725, U.S.

^4^Department of Geosciences, Boise State University, Boise, Idaho 83725, U.S.

^5^Department of Geography, University of Utah, Salt Lake City, Utah, 84112, U.S.

^6^Department of Geography, University of Idaho, Moscow, Idaho, 83844, U.S.

^7^Department of Civil and Environmental Engineering, University of California, Irvine, California 92697, U.S.

^8^Department of Community and Environmental Health, Boise State University, Boise, Idaho, 83725, U.S.

*Correspondence to*: Mojtaba Sadegh ([mojtabasadegh@boisestate.edu](mailto:mojtabasadegh@boisestate.edu))

**Table S1. Survey questions and response rate to each question for 614 in-person and 1,623 online participants.**

| **Question number** | **Question text** | **Response rate** | |
| --- | --- | --- | --- |
|  |  | **In-person (%)** | **Online (%)** |
| **Demographics** | | | |
| 2 | What is your age? | 98.53 | 95.30 |
| 3 | Which gender do you identify with? | 99.67 | 95.65 |
| 4 | What racial or ethnic group best describes you? | 99.19 | 95.42 |
| 5 | What is the ZIP Code where you currently live? | 98.53 | 94.56 |
| 6 | What is the highest degree or level of school you completed? If currently enrolled, highest degree received. | 98.70 | 95.59 |
| 7 | What is your total household income, including income from all members of your family, in 2017 before taxes? This figure should include salaries, wages, pensions, dividends, interest, and all other income. | 93.97 | 93.01 |
| Activity | | | |
| 8 | Would you say that in general your health is: | 97.88 | 94.73 |
| 9 | During summer of 2018, have you engaged in any outdoor activities, such as hiking, biking, fishing, gardening, running, or any other outdoor activity? | 97.72 | 94.62 |
| 10 | During the summer of 2018, how often would you say you’ve engaged in the outdoor activities you’ve listed above? | 92.67 | 87.23 |
| Air Quality Notification | | | |
| 11 | During the summer of 2018, have you ever received an air quality notification message suggesting you avoid outside activity? | 97.56 | 94.33 |
| 12 | Do you ever seek out information related to wildfire and smoke notifications? | 97.56 | 94.39 |
| 13 | Which source do you use to find wildfire smoke notifications? [Check all that apply] | 91.86 | 81.56 |
| 14 | In a smoky week in summer 2018, about how many days did you look online (either on a computer, tablet, or smartphone) for smoke-related information, such as air quality, smoke forecasts, or health notices? | 89.90 | 81.67 |
| 15 | During summer of 2018, did you *ever* reduce your outside activities due to wildfire smoke? | 99.02 | 91.41 |
| 16 | During summer of 2018, think of the *longest* period of consecutive days you reduced or eliminated your outdoor activities due to a smoke event. How many consecutive days did you reduce or eliminate activity? | 98.37 | 93.07 |
| 17 | What is the *minimum* air quality index rating that would cause you to reduce your outdoor activity on a particular day? | 93.32 | 93.18 |
| 18 | What is the *minimum* air quality index rating that would cause you to eliminate your outdoor activity on a particular day? | 94.30 | 93.24 |
| 19 | If you decided to limit your outdoor activity during a smoke event, what type of information motivated your decision to do so? [Check all that apply] | 93.00 | 87.97 |
| 20 | What type of message could motivate / motivated you to take action to mitigate the risk of issues related to wildfire smoke, such as staying indoors or leaving the area? [Check all that apply] | 96.91 | 89.18 |
| 21 | What was the *content* of the message(s) that motivated you to take this action to mitigate the negative health impacts of wildfire smoke? [Check all that apply] | 85.99 | 84.88 |
| 22 | When would receiving a smoke warning message be most likely to impact your decision to limit or avoid outdoor activities *that same day?* | 94.95 | 90.38 |
| 23 | Will you take preventive actions to reduce smoke-related health impacts in the future? | 94.79 | 91.35 |
| Natural Hazard | | | |
| 24 | Do you consider wildfire smoke events a natural hazard? | 96.91 | 91.12 |
| 25 | As a public health threat, are wildfire smoke events more important, less important, or about as important as other natural disasters, such as hurricanes or tornadoes? | 95.11 | 90.95 |
| 26 | Would you consider evacuating your home only because of the wildfire smoke? | 96.91 | 90.95 |
| Health | | | |
| 27 | Have you, or anyone in your household, experienced wildfire smoke-related illness? | 97.23 | 90.61 |
| 28 | Did you have any of the following symptoms during or a few days after one of the smoke events in the summer of 2018 in the Boise area / Treasure Valley? [Check all that apply] | 93.16 | 81.39 |
| 29 | Did you use/do any of the following to help with any symptoms during the smoke event? [Check all that apply] | 83.55 | 54.98 |

**Table S2. Summary information about the location and time of in-person surveys in the Boise, Idaho Metropolitan area.**

| **Location** | **Date** | **Time** | **Collected Surveys** |
| --- | --- | --- | --- |
| Julia Davis Park - Boise | 8/28/2018 | 5:30-8:00pm | 40 |
| Alive After 5 - Public Concert - Boise | 8/29/2018 | 5:30-8:30pm | 58 |
| Kristin Armstrong & Ann Morrison Park - Boise | 8/30/2018 | 5:30-8:00pm | 44 |
| Movie in the Park - Meridian | 8/31/2018 | 6:30-9:00pm | 29 |
| Night Glow - Public Event, Balloon Classic - Boise | 8/31/2018 | 5:30-8:00pm | 102 |
| Farmers Market - Meridian | 9/1/2018 | 10:00am - noon | 25 |
| Julia Davis Park - Boise | 9/1/2018 | noon - 12:30pm | 4 |
| Downtown Eagle & Eagle Island State Park - Eagle | 9/2/2018 | 1:00-3:30pm | 21 |
| Guerber Park - Eagle | 9/4/2018 | 6:00-7:30pm | 30 |
| Bernie Fisher Park and Kuna Middle School - Kuna | 9/5/2018 | 6:00-7:30pm | 31 |
| Ester Simplot Park - Boise | 9/6/2018 | 6:00-7:00pm | 15 |
| Kristin Armstrong Park - Boise | 9/7/2018 | 5:30-6:30pm | 24 |
| Farmers Market & Lakeview Park - Nampa | 9/8/2018 | 9:30-1:00pm | 34 |
| BSU Home Football Game - Boise | 9/8/2018 | 4:30-8:00pm | 90 |
| Farmers Market - Caldwell | 9/12/2018 | 3-7:00pm | 37 |
| Hyde Park Street Fair - Boise | 9/15/2018 | 12:30-2:00pm | 24 |
| Boise | varied |  | 6 |

**Table S3. Race Demographics of Boise, ID, According to the 2017 American Community Survey (ACS) versus Age Demographics in Our Collected Data Set**

| **Race** | ACS report | In-person collected data | Online collected data |
| --- | --- | --- | --- |
| **White alone** | 82.60% | 88.8% | 83.5% |
| **Black or African American alone** | 1.80% | 0.7% | 0.6% |
| **American Indian and Alaska Native alone** | 0.60% | 1.6% | 0.5% |
| **Asian alone** | 3.30% | NA^*^ | NA^*^ |
| **Native Hawaiian and Other Pacific Islander alone** | 0.06% | NA^*^ | NA^*^ |
| **Some other race alone** | 0.10% |  | 3% |
| **Two or more races:** | 2.90% | NA^*^ | NA^*^ |
| **Hispanic or Latino:** | 8.70% | 5.7% | 5.7% |

*Cannot be reported due to difference in definition

Source: <https://factfinder.census.gov/faces/tableservices/jsf/pages/productview.xhtml?src=bkmk>

For graphical presentation see: <http://worldpopulationreview.com/us-cities/boise-population/>

Table S4. Age Demographics in Boise, Idaho According to American Community Survey (ACS) 2017 versus Age Demographics in Our Collected Data Set

|  | ACS report | In-person collected data | Online collected data |
| --- | --- | --- | --- |
| **5 to 9 years** | 6.20% | NA | NA |
| **10 to 14 years** | 6.40% | NA | NA |
| **15 to 19 years** | 6.80% | 1.1% (only 18-19) | 11.2% (only 18-19) |
| **20 to 24 years** | 8.00% | 10.1% | 16.6% |
| **25 to 29 years** | 8.00% | 13.4% | 9.5% |
| **30 to 34 years** | 7.40% | 12.2% | 8.9% |
| **35 to 39 years** | 7.30% | 12.4% | 8.1% |
| **40 to 44 years** | 6.40% | 10.9% | 9.5% |
| **45 to 49 years** | 6.20% | 9.6% | 7.2% |
| **50 to 54 years** | 6.30% | 4.6% | 7.8% |
| **55 to 59 years** | 6.40% | 7.2% | 6.8% |
| **60 to 64 years** | 5.80% | 8.0% | 5.8% |
| **65 to 69 years** | 4.80% | 4.9% | 2.6% |
| **70 to 74 years** | 3.10% | 2.9% | 0.9% |
| **75 to 79 years** | 1.90% | 0.8% | 0.3% |
| **80 to 84 years** | 1.50% | 0.3% | 0% |
| **85 years and over** | 1.90% | 0% | 0% |

Source: <https://factfinder.census.gov/faces/tableservices/jsf/pages/productview.xhtml?src=bkmk>

For graphical presentation see: <http://worldpopulationreview.com/us-cities/boise-population/>

Table S5: Education Demographics of Individuals of 25 Years and Older in Boise, ID, According to the 2017 ACS versus Age Demographics in Our Collected Data Set

|  | ACS report | In-person collected data | Online collected data |
| --- | --- | --- | --- |
| **Less than 9th grade** | 1.70% | NA^*^ | NA^*^ |
| **9th to 12th grade, no diploma** | 3.90% | NA^*^ | NA^*^ |
| **High school graduate (includes equivalency)** | 20.70% | 11.1% | NA^*^ |
| **Some college, no degree** | 24.10% | 24.6% | NA^*^ |
| **Associate's degree** | 8.70% | 7.8% | NA^*^ |
| **Bachelor's degree** | 26.60% | 37.0% | NA^*^ |

*Cannot be reported due to difference in definition

Source: <https://factfinder.census.gov/faces/tableservices/jsf/pages/productview.xhtml?src=bkmk>

For graphical presentation see: <http://worldpopulationreview.com/us-cities/boise-population/>

Table S6: Air Quality Index (AQI) Rating (EPA 2016)

**
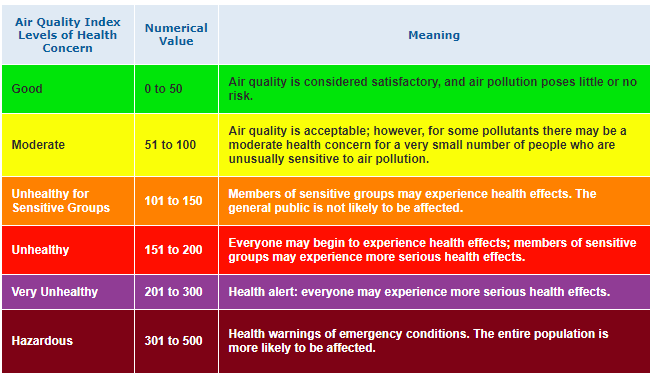
**

Source:

United States Government, AIRNOW. 2016. “Air Quality Index (AQI) Basics.” https://airnow.gov/index.cfm?action=aqibasics.aqi (November 2, 2018).

**Figure S1: Daily AQI for Boise, Idaho between 1999 to 2018**

**Figure S2: Daily Boise AQI Fire Season - July through September**

**Figure S3: Daily Boise AQI Inversion Months - November through February**

**Figure S4: Daily Boise AQI Non-fire and Non-inversion Months - March through June, and October**

**
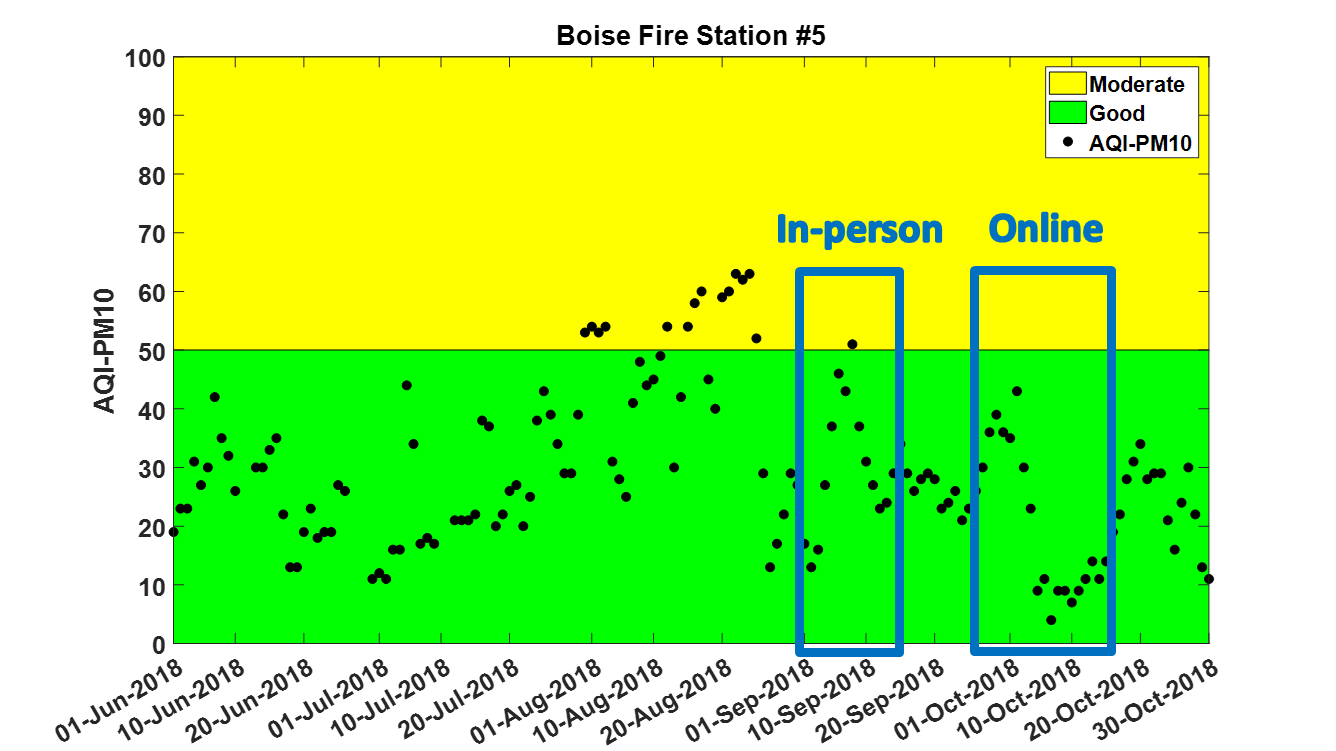
**

**Figure S5: Summer 2018 Daily Boise AQI Values**


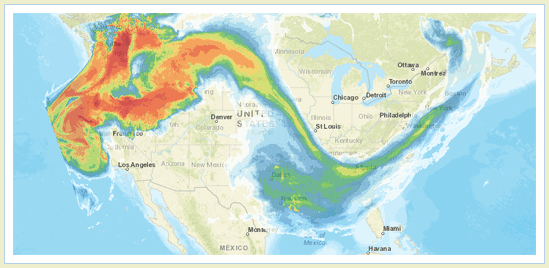


**Figure S6: Wildfire Smoke and Air Quality Maps on August 23, 2018 Provided by NOAA's Smoke Forecast.**

**
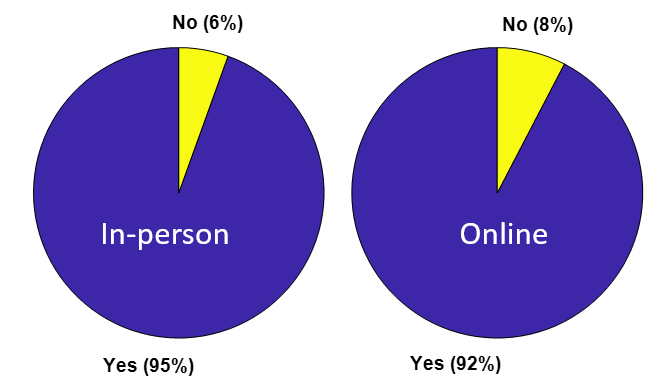
**

**Figure S7: Outdoor Activity Participation**

**
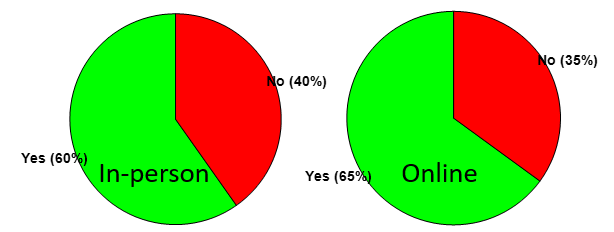
**

**Figure S8: Participants Received Air Quality Notifications to Avoid Outside Activity**

**
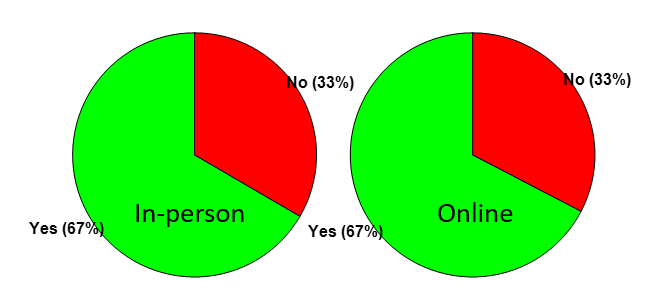
**

**Figure S9: Participants Sought Air Quality Notifications Related to Wild-fire and Smoke**

**
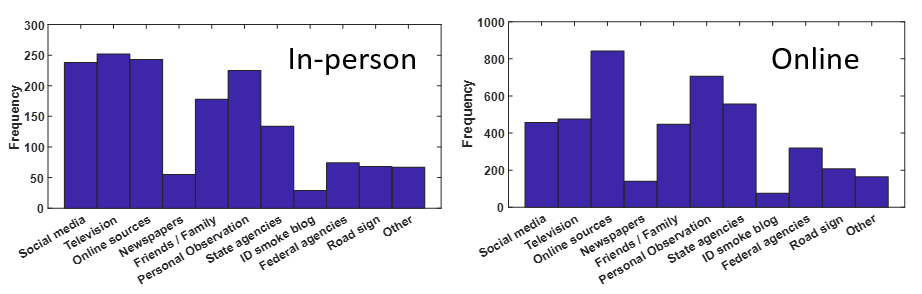
**

**Figure S10: Participants Communication Sources Regarding Air Quality**

**
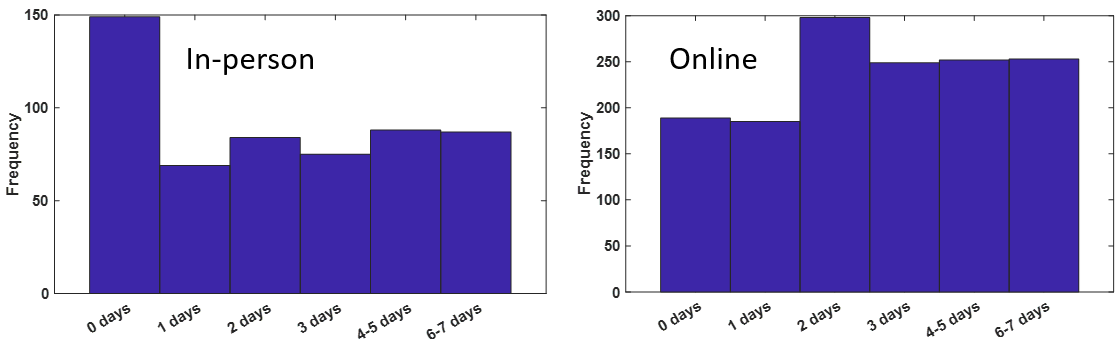
**

**Figure S11: Number of Days per Week Participants Sought On-line Air Quality Information**

**
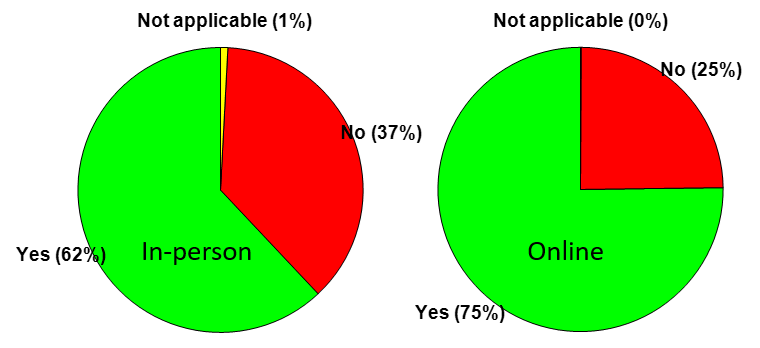
**

**Figure S12: Participants Reduction in Outdoor Activities Due to Air Quality**

**
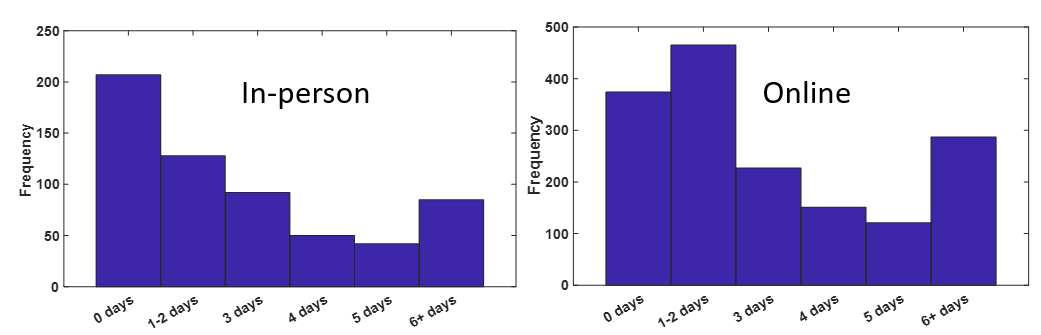
**

**Figure S13: Number of Days Participants Reduced/Eliminated Outdoor Activities**

**
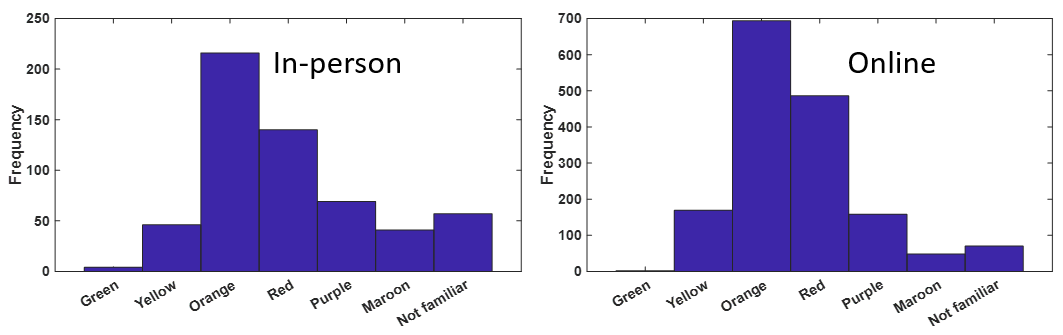
**

**Figure S14: AQI Rating Level Causing Participants to Reduce Outdoor Activities**

**
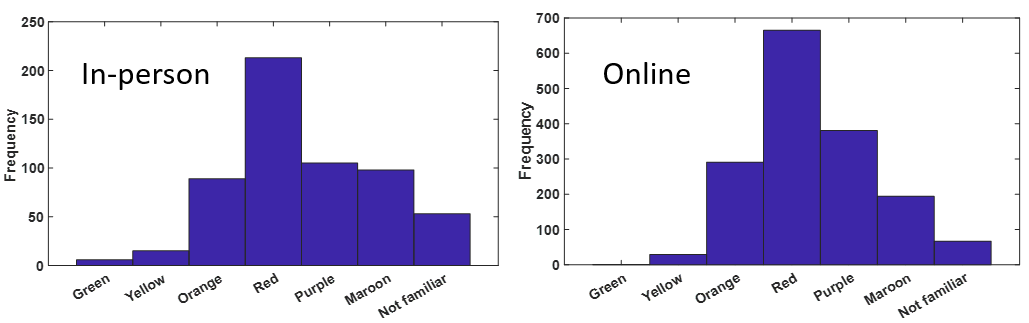
**

**Figure S15: AQI Rating Level Causing Participants to Eliminate Outdoor Activities**

**
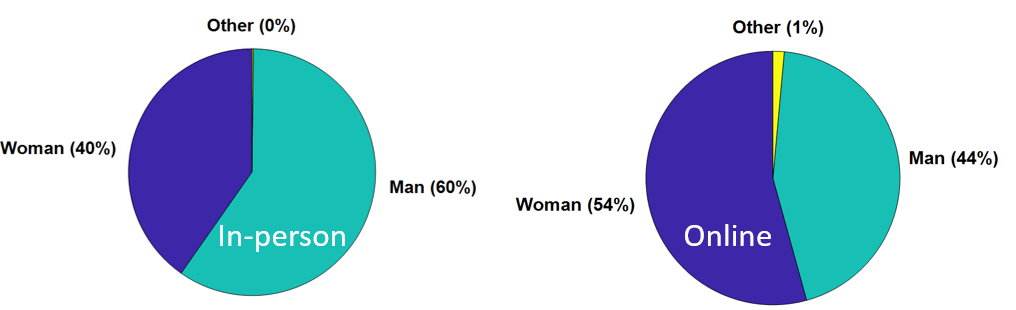
**

**Figure S16: Gender of Participants Unfamiliar with AQI Rating Levels**

**
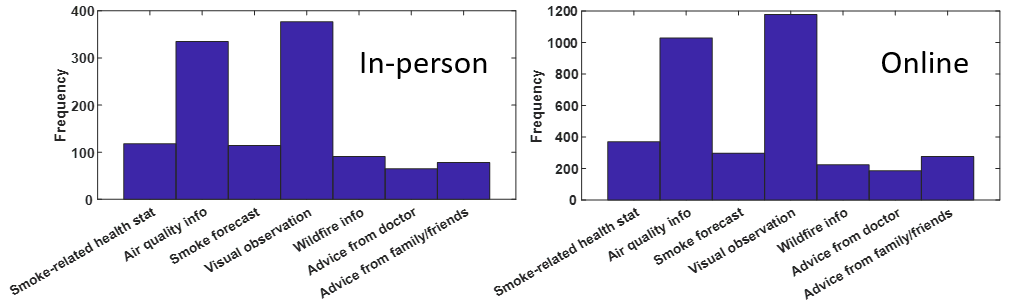
**

**Figure S17: Factors Causing Participants to Limit Outdoor Activities**

**
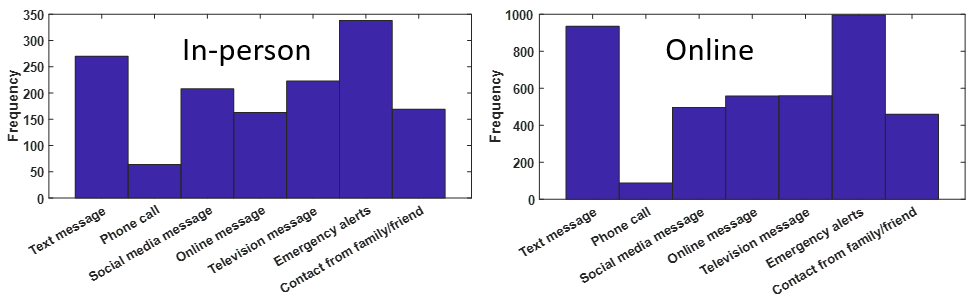
**

**Figure S18: Type of Message Motivating Participant to Take Action to Reduce Risk**

**
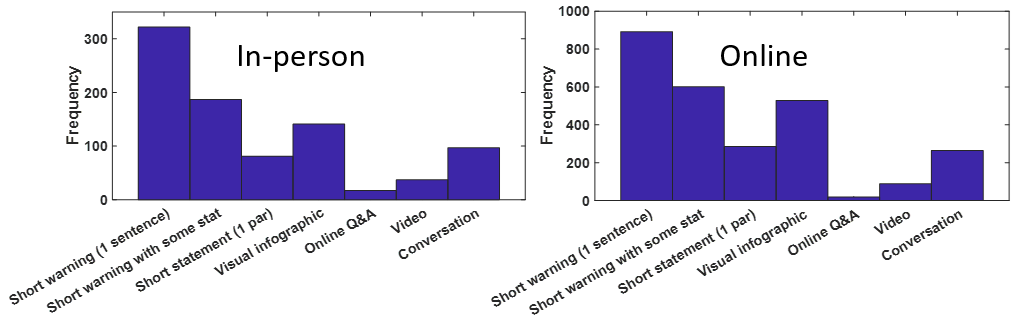
**

**Figure S19: Message Content Motivating Participant to Take Action to Reduce Risk**

**
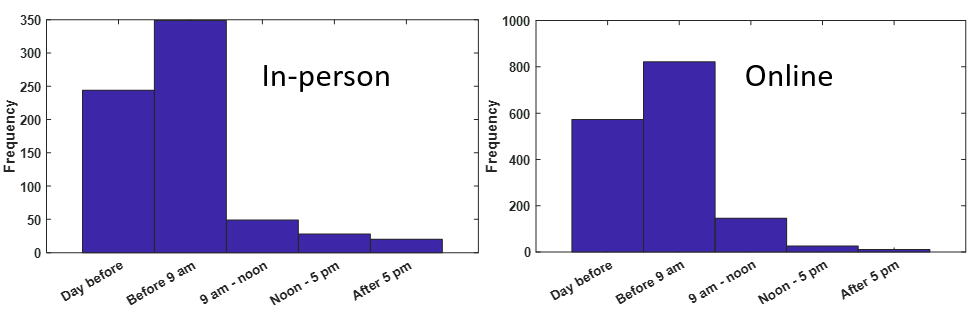
**

**Figure S20: Timing of Warning Messages Encouraging Participants to Limit/avoid Outside Activities**

**
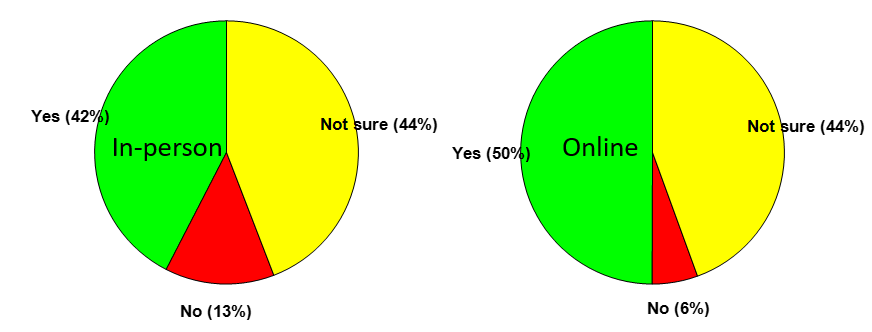
**

**Figure S21: Participants Would Take Preventative Measures in the Future**

**
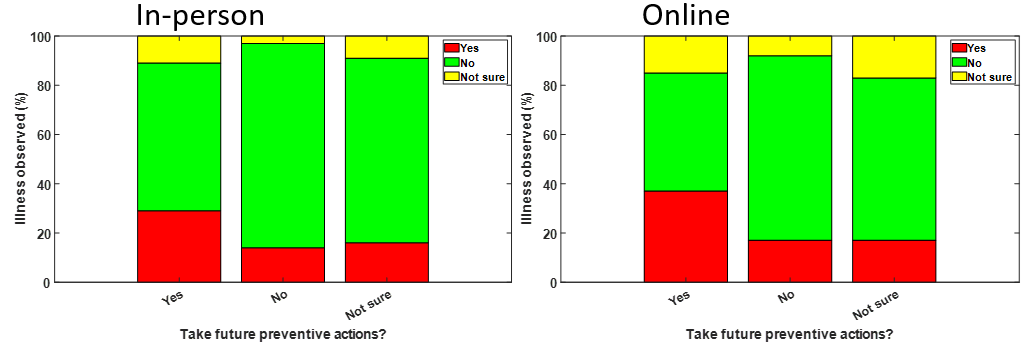
**

**Figure S22: Participants Experiencing Air Quality Related Illness Will Take Preventative Measures in Future**

**
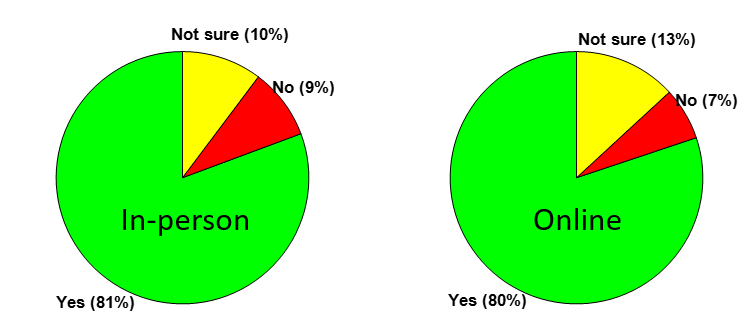
**

**Figure S23: Participants Consider Wildfire Smoke a Natural Disaster**

**
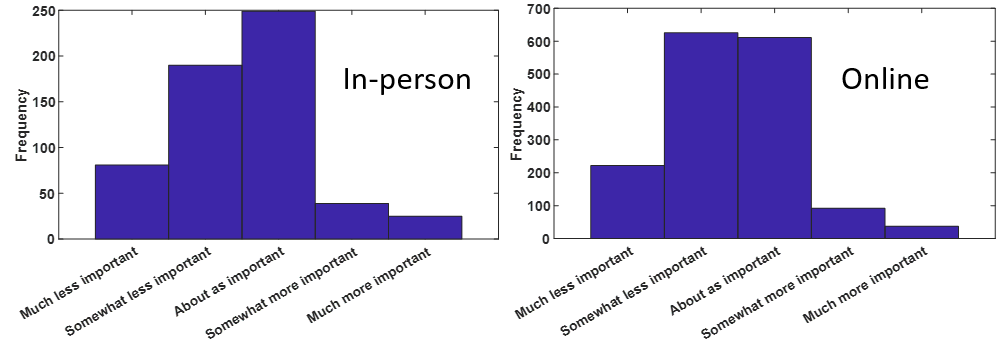
**

**Figure S24: Participants Comparison of Wildfire Smoke to Other Natural Disasters**

**
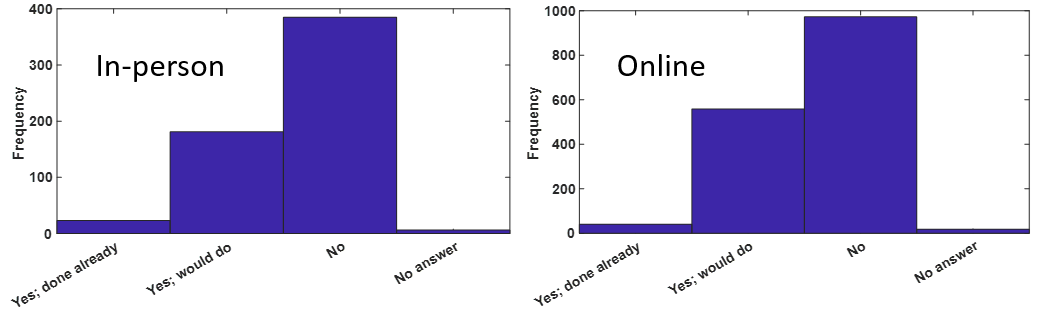
**

**Figure S25: Participants Consideration of Evacuating Home During Wildfire Smoke Event**

**
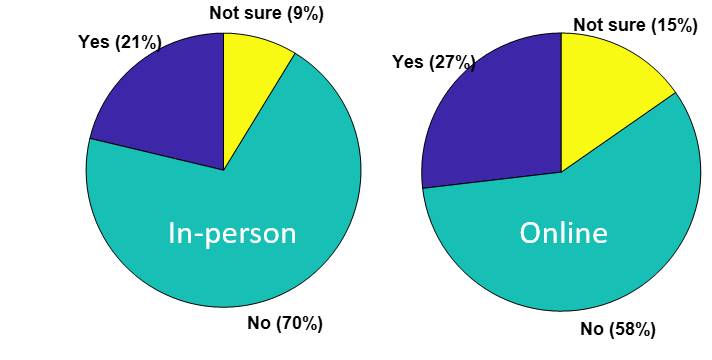
**

**Figure S26: Participants Experiencing Wildfire Smoke-Related Illnesses**

**
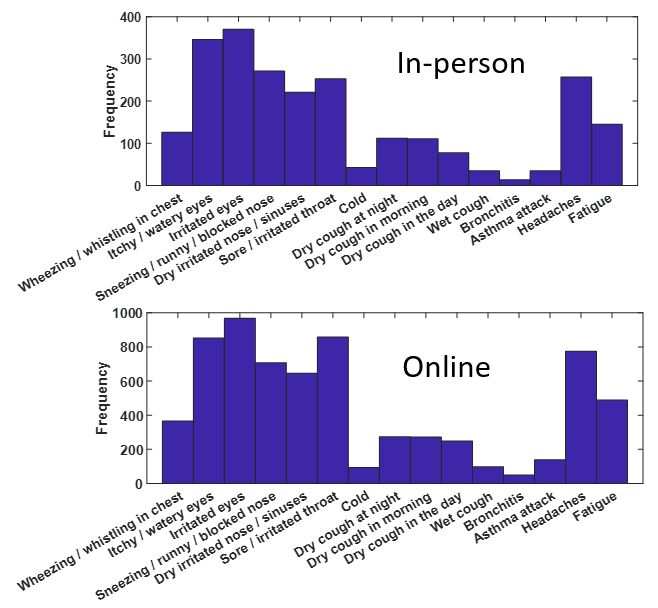
**

**Figure S27: Symptoms Participants Experience during Wildfire Smoke Event**

**
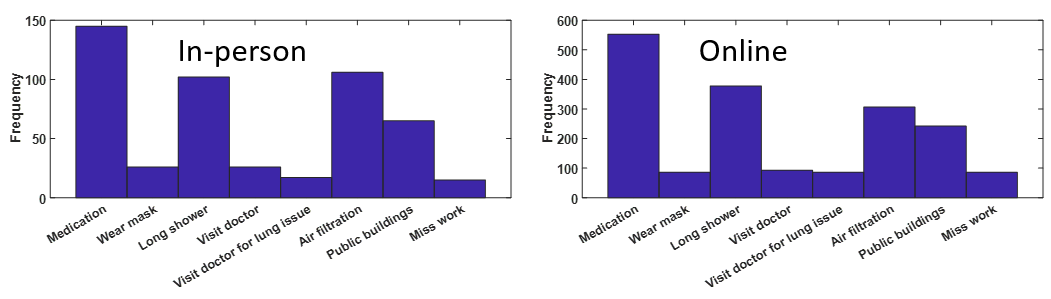
**

**Figure S28: How Participants Mitigated Symptoms during Wildfire Smoke Event**

**
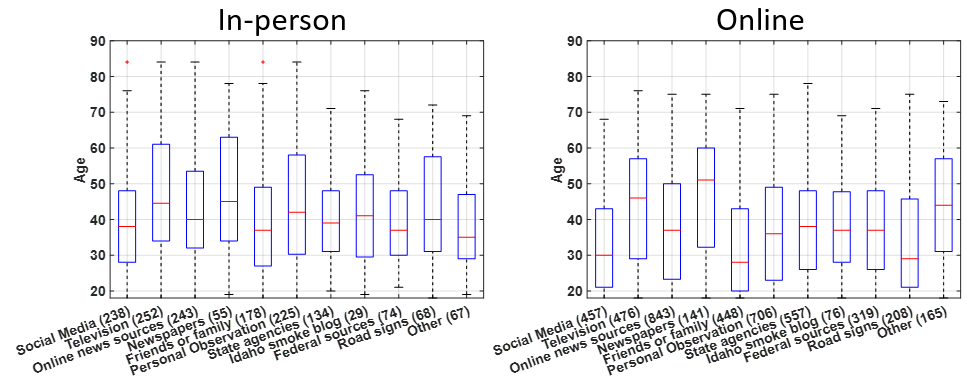
**

**Figure S29: Age and Source of Air Quality**

**
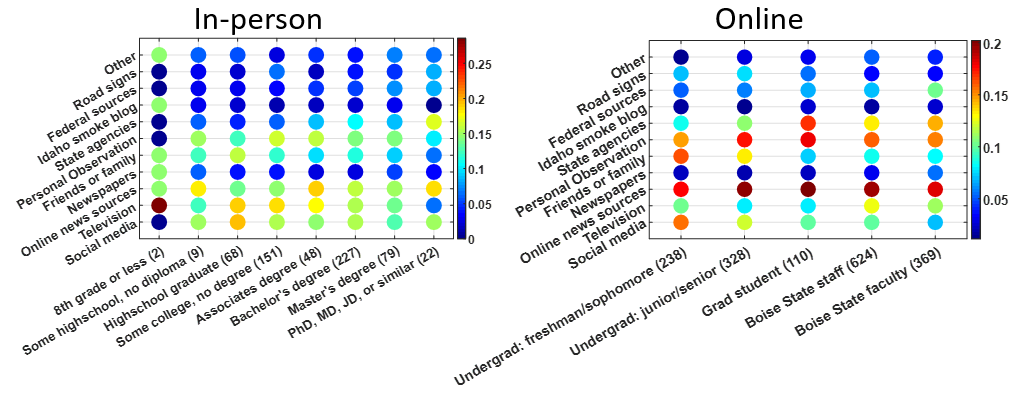
**

Figure S30: Education and Source of Air Quality


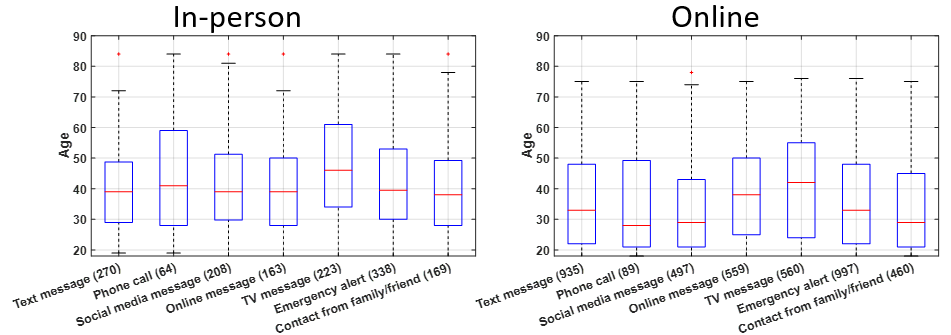
 Figure S31: Age and Message Medium

**
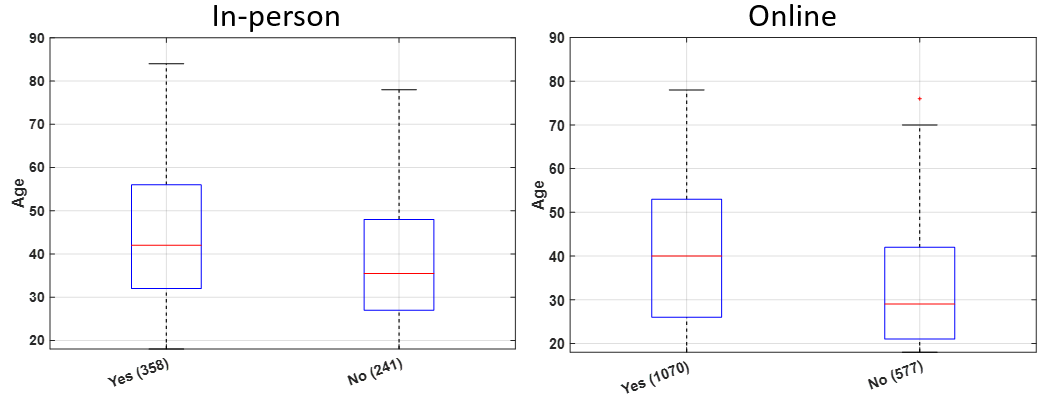
**

**Figure S32: Age and Received Air Quality Information**

**
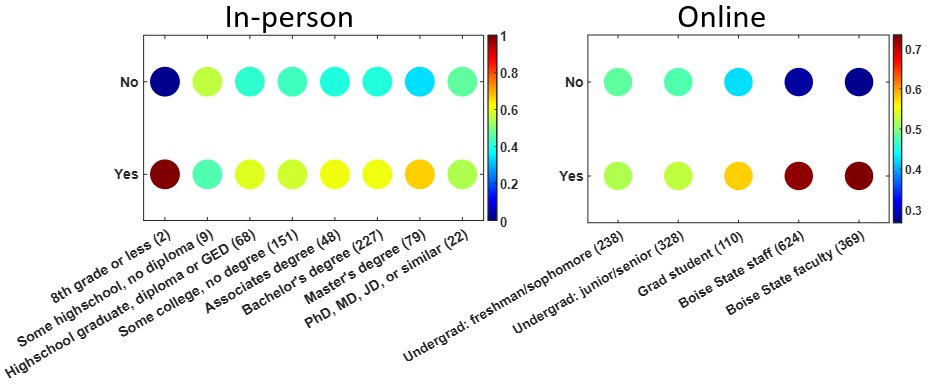
**

**Figure S33: Education and Received Air Quality Information**

**
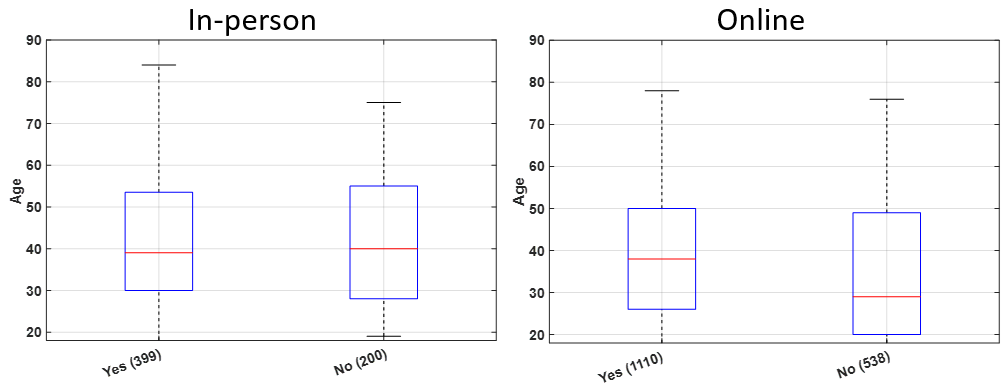
**

**Figure S34: Age and Sought Air Quality Information**


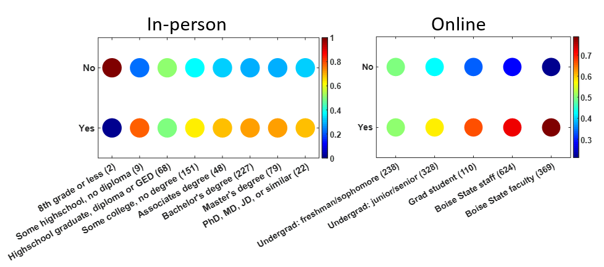


**Figure S35: Education and Sought Air Quality Information**

**
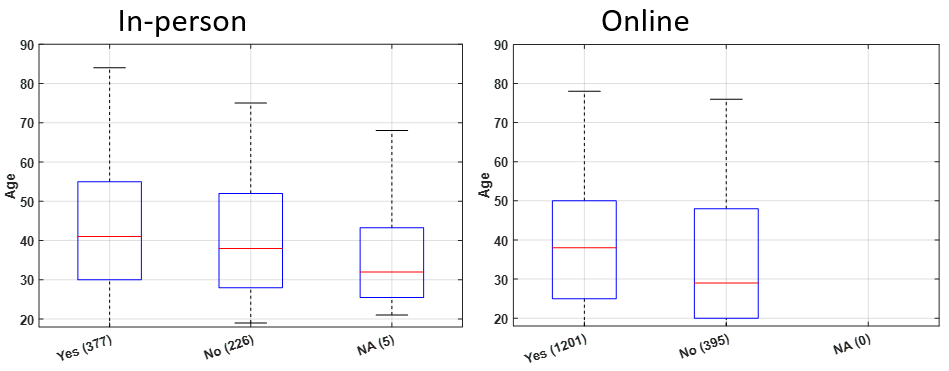
**

Figure S36: Age and Reduced Activities


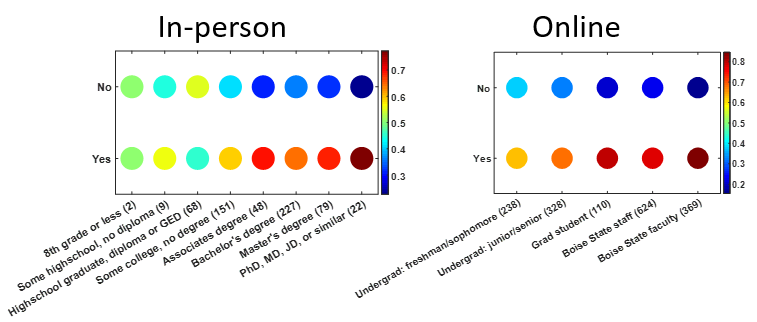
 Figure S37: Education and Reduced Activities


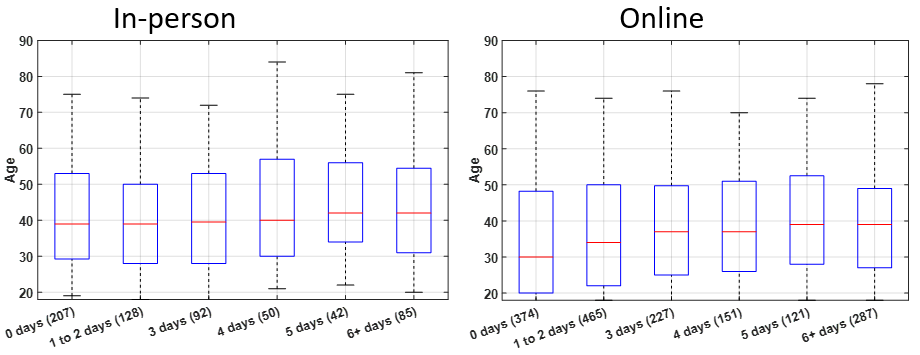


**Figure S38: Age and Number of Days Reduced Activities**

**
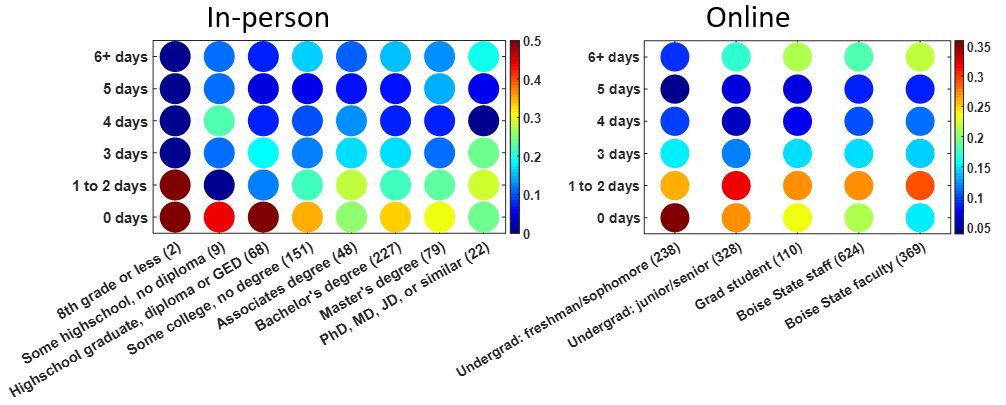
**

**Figure S39: Education and Number of Days Reduced Activities**
