# Supplementary material for: A dataset on human perception of and response to wildfire smoke
Source: Sci Data. 2019 Oct 24;6:229. doi: 10.1038/s41597-019-0251-y (PMC6813346; doi:10.1038/s41597-019-0251-y)
Supplement: Supplementary file 2 — Appendix A. [file 41597_2019_251_MOESM2_ESM.pdf]

Appendix A: Survey Questionnaire

Mojtaba Sadegh and Mariah Fowler Consent Form

Title: Smoke Waves in the Treasure Valley

Alternative title: Human Response to Wildfire Smoke Survey

Study Information Sheet

The study is being conducted by Mojtaba Sadegh, assistant professor, and Mariah Fowler, graduate student of Civil Engineering, at Boise State University. The purpose of this study is strictly for research purposes. The researchers are not affiliated in any way with any organization other than Boise State University.

The purpose of this study is to assess human response to wildfire smoke. During the study, you will answer some survey questions and read a brief text. This survey should take you less than 10 minutes to complete. You must be at least 18 years old to complete this survey.

This study involves no foreseeable risks. You may discontinue the study at any time. Your responses are completely anonymous and cannot be linked to you in any way.

For this research project, we are requesting demographic information. Though it is unlikely, it is possible that the combined answers to these questions may make an individual person identifiable. The researchers will make every effort to protect your confidentiality. However, if you are uncomfortable answering any of these questions, you may leave them blank.

CONTACTS FOR QUESTIONS OR PROBLEMS

Contact Information: If you should have any questions about this research study, please contact Mojtaba Sadegh at 208-426-3774 or mojtabasadegh@boisestate.edu. For additional information about your rights as a research participant in this study, please feel free to contact the Boise State University Institutional Review Board Office. You may reach the board office between 8:00 AM and 5:00 PM, Monday through Friday, by calling (208) 426-5401 or by writing: Institutional Review Board, Office of Research Compliance, Boise State University, 1910 University Dr., Boise, ID 83725-1138.

In consideration of all of the above, I give my consent to participate in this research study.

In consideration of all of the above, I DO NOT consent to participate in this research study. [EXIT IF NO CONSEN]

Date:

DEMOGRAPHIC DATA **Survey location:**

If you are uncomfortable answering any of these questions, you may leave them blank.

1. Do you consent to participate in this research study?
   - ☐ Yes
   - ☐ No – If you do not consent to participate, please do not complete this survey

2. What is your age?

   Fill in the blank _______

3. Which gender do you identify with?
   - ☐ A woman
   - ☐ A man
   - ☐ Other _____________

4. What racial or ethnic group best describes you?
   - ☐ White / Caucasian
   - ☐ Hispanic or Latino
   - ☐ Black or African American
   - ☐ Native American / American Indian or Alaskan Native
   - ☐ Asian / Pacific Islander
   - ☐ Other _______

5. What is the ZIP Code where you currently live?

   Fill in the blank _______

6. What is the highest degree or level of school you completed? If currently enrolled, highest degree received.
   - ☐ 8th grade or less
   - ☐ Some high school, no diploma
   - ☐ High school graduate, diploma or GED
   - ☐ Some college, no degree
   - ☐ Associates degree
   - ☐ Bachelor's degree
   - ☐ Master's degree
   - ☐ Ph.D, M.D., J.D. or similar

7. What is your total household income, including income from all members of your family, in 2017 before taxes? This figure should include salaries, wages, pensions, dividends, interest, and all other income.
   - ☐ $25,000 or less
   - ☐ $25,000 to $49,999
   - ☐ $50,000 to $74,999
   - ☐ $75,000 to $99,999
   - ☐ $100,000 or more

ACTIVITY DATA

8. Would you say that in general your health is:
   - ☐ Excellent
   - ☐ Good
   - ☐ Fair
   - ☐ Poor

9. During summer of 2018, have you engaged in any outdoor activities, such as hiking, biking, fishing, gardening, running, or any other outdoor activity?
   - ☐ Yes – Please list the activities ___________________
   - ☐ No (skip to question 11)

10. During the summer of 2018, how often would you say you've engaged in the outdoor activities you've listed above?
    - ☐ Daily
    - ☐ A few times per week
    - ☐ Once per week
    - ☐ Less than once per week, but more than once per month
    - ☐ Rarely – A few times during the summer
    - ☐ Never

AIR QUALITY NOTIFICATION

11. During the summer of 2018, have you ever received an air quality notification message suggesting you avoid outside activity?
    - ☐ Yes
    - ☐ No
12. Do you ever seek out information related to wildfire and smoke notifications?
    - ☐ a. Yes
    - ☐ b. No (if you answered no to both questions 11 and 12, please skip to question 15)

13. Which source do you use to find wildfire smoke notifications? [Check all that apply]
    - ☐ Social media (like Facebook, Instagram, Twitter)
    - ☐ Television
    - ☐ Online news sources
    - ☐ Newspapers
    - ☐ Friends or Family
    - ☐ Personal observation
    - ☐ State agencies such as Department of Environmental Quality website
    - ☐ Idaho Smoke Information Blog
    - ☐ Federal sources such as AIRnow.gov website
    - ☐ Dynamic road sign displays (such as the signs on I-184 or I-84)
    - ☐ Other _______

14. In a smoky week in summer 2018, about how many days did you look online (either on a computer, tablet, or smartphone) for smoke-related information, such as air quality, smoke forecasts, or health notices?
    - ☐ 0 days
    - ☐ 1 days
    - ☐ 2 days
    - ☐ 3 days
    - ☐ 4-5 days
    - ☐ 6-7 days.

15. During summer of 2018, did you *ever* reduce your outside activities due to wildfire smoke?
    - ☐ Yes
    - ☐ No
    - ☐ Not applicable

16. During summer of 2018, think of the *longest* period of consecutive days you reduced or eliminated your outdoor activities due to a smoke event. <u>How many consecutive days</u> did you reduce or eliminate activity?
    - ☐ 0 days
    - ☐ 1 to 2 days
    - ☐ 3 days
    - ☐ 4 days
    - ☐ 5 days
    - ☐ 6 days and more

17. What is the *minimum* air quality index rating that would cause you to <u>reduce your outdoor activity</u> on a particular day?
    - ☐ Green – Good
    - ☐ Yellow – Moderate
    - ☐ Orange – Unhealthy for Sensitive Groups
    - ☐ Red – Unhealthy
    - ☐ Purple – Very Unhealthy
    - ☐ Maroon – Hazardous
    - ☐ I am not familiar with this rating

18. What is the *minimum* air quality index rating that would cause you to <u>eliminate your outdoor activity</u> on a particular day?
    - ☐ Green – Good
    - ☐ Yellow – Moderate
    - ☐ Orange – Unhealthy for Sensitive Groups
    - ☐ Red – Unhealthy
    - ☐ Purple – Very Unhealthy
    - ☐ Maroon – Hazardous
    - ☐ I am not familiar with this rating

19. If you decided to limit your outdoor activity during a smoke event, what type of information motivated your decision to do so? [Check all that apply]
   - ☐ Smoke-related health problem statistics
   - ☐ Air quality information
   - ☐ Smoke forecasts
   - ☐ Your own visual observation (seeing the smoke outside)
   - ☐ Wildfire information
   - ☐ Advice from your doctor
   - ☐ Advice from family and friends

20. What type of message could motivate / motivated you to take action to mitigate the risk of issues related to wildfire smoke, such as staying indoors or leaving the area? [Check all that apply]
   - ☐ Text message
   - ☐ Phone call
   - ☐ Social media message
   - ☐ Online message
   - ☐ Message seen on television
   - ☐ Emergency alerts
   - ☐ Contact from family or friends

21. What was the *content* of the message(s) that motivated you to take this action to mitigate the negative health impacts of wildfire smoke? [Check all that apply]
   - ☐ A short message warning about the risk (1 line of text)
   - ☐ A short message warning about the risk that included health or other statistics
   - ☐ A short statement (roughly 1 paragraph)
   - ☐ A visual infographic or picture
   - ☐ An online Q&A session (Facebook Live or Instagram Live)
   - ☐ A video
   - ☐ A conversation (either online, via phone, or in person)

22. When would receiving a smoke warning message be most likely to impact your decision to limit or avoid outdoor activities *that same day?*
   - ☐ I would prefer to know the day before
   - ☐ Early morning (before 9 AM)
   - ☐ Late morning (9 AM – noon)
   - ☐ Afternoon (Noon – 5 PM)
   - ☐ Evening (5 PM or later)

23. Will you take preventive actions to reduce smoke-related health impacts in the future?
   - ☐ Yes – Please list the actions you might take ______________
   - ☐ No
   - ☐ Not sure

NATURAL HAZARD QUESTIONS

24. Do you consider wildfire smoke events a natural hazard?
    - ☐ Yes
    - ☐ No
    - ☐ Not sure

25. As a public health threat, are wildfire smoke events more important, less important, or about as important as other natural disasters, such as hurricanes or tornadoes?
    - ☐ Much less severe/important
    - ☐ Somewhat less severe/important
    - ☐ About as severe/important
    - ☐ Somewhat more severe/important
    - ☐ Much more severe/important

26. Would you consider evacuating your home only because of the wildfire smoke?
    - ☐ Yes, I have done this in the past.
    - ☐ Yes, I would consider it
    - ☐ No
    - ☐ Prefer not to answer

HEALTH QUESTIONS

27. Have you, or anyone in your household, experienced wildfire smoke-related illness?
    - ☐ Yes
    - ☐ No
    - ☐ Not sure

28. Did you have any of the following symptoms during or a few days after one of the smoke events in the summer of 2018 in the Boise area / Treasure Valley? [Check all that apply]
    - ☐ Wheezing or whistling in the chest
    - ☐ Itchy or watery eyes
    - ☐ Irritated eyes
    - ☐ Sneezing or a runny or blocked nose
    - ☐ Dry irritated nose / sinuses
    - ☐ A sore or irritated throat
    - ☐ A cold
    - ☐ A dry cough at night
    - ☐ A dry cough first thing in the morning
    - ☐ A dry cough at other times of the day
    - ☐ A wet cough (congestion in the chest or phlegm production)
    - ☐ Bronchitis
    - ☐ An asthma attack
    - ☐ Headaches
    - ☐ Fatigue

29. Did you use/do any of the following to help with any symptoms during the smoke event? [Check all that apply]

- ☐ Take medication
- ☐ Wear a mask to protect your lungs
- ☐ Take long showers
- ☐ Visit a doctor or nurse
- ☐ Visit a doctor’s office for asthma or smoke-related lung issues
- ☐ Use a personal air filtration system in your home or office
- ☐ Go to buildings that have air filtration systems like the mall or public library
- ☐ Miss work due to health problems
