# Supplementary figures and images for: A dataset on human perception of and response to wildfire smoke
Source: Sci Data. 2019 Oct 24;6:229. doi: 10.1038/s41597-019-0251-y (PMC6813346; doi:10.1038/s41597-019-0251-y)

# Appendix B: Internal Review Board Approval
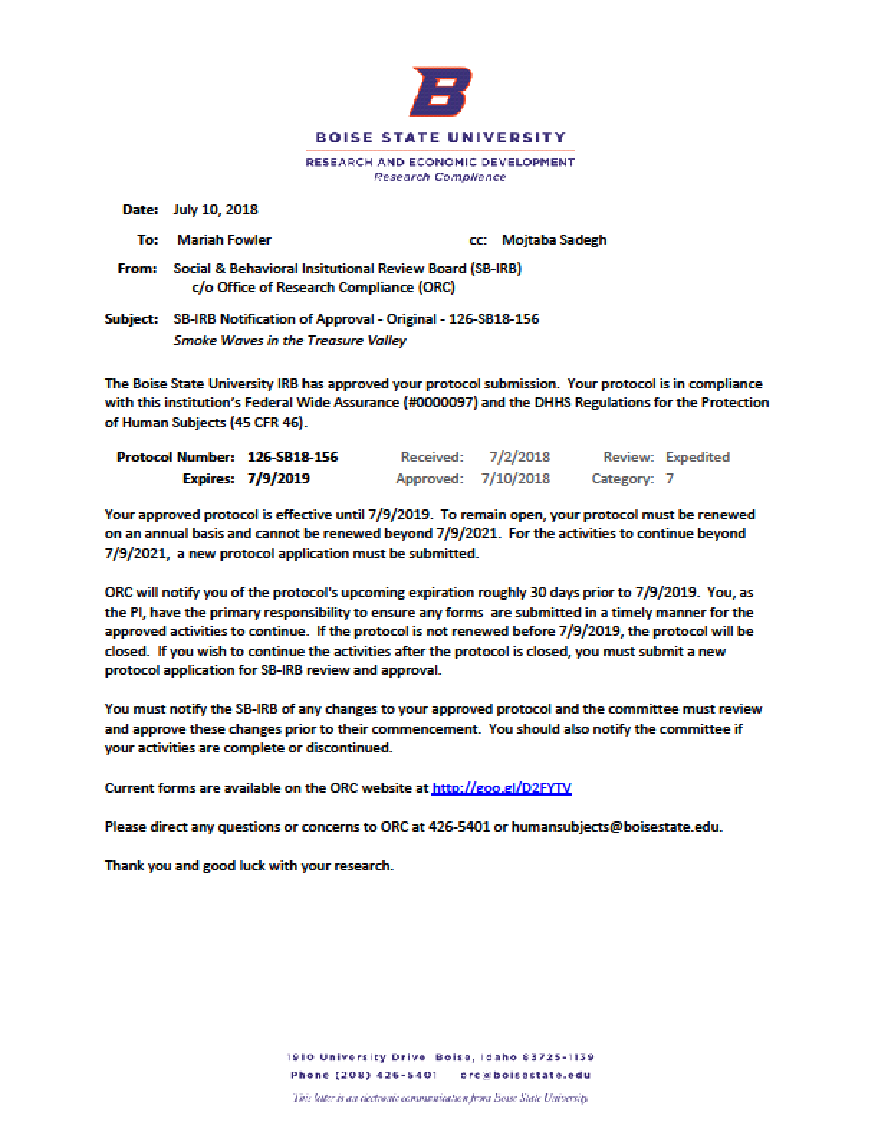

Supplement: Supplementary file 3 — Appendix B. [file 41597_2019_251_MOESM3_ESM.docx]
